# Effects of Quadriceps Muscle Fatigue on Stiff-Knee Gait in Patients with Hemiparesis

**DOI:** 10.1371/journal.pone.0094138

**Published:** 2014-04-09

**Authors:** Julien Boudarham, Nicolas Roche, Didier Pradon, Eric Delouf, Djamel Bensmail, Raphael Zory

**Affiliations:** 1 GRCTH, EA4497, CIC-IT 805, CHU Raymond Poincaré, Garches, France; 2 LAMHESS, EA 6309, University of Nice Sophia Antipolis, Nice, France; University of Sao Paulo, Brazil

## Abstract

The relationship between neuromuscular fatigue and locomotion has never been investigated in hemiparetic patients despite the fact that, in the clinical context, patients report to be more spastic or stiffer after walking a long distance or after a rehabilitation session. The aim of this study was to evaluate the effects of quadriceps muscle fatigue on the biomechanical gait parameters of patients with a stiff-knee gait (SKG). Thirteen patients and eleven healthy controls performed one gait analysis before a protocol of isokinetic quadriceps fatigue and two after (immediately after and after 10 minutes of rest). Spatiotemporal parameters, sagittal knee and hip kinematics, rectus femoris (RF) and vastus lateralis (VL) kinematics and electromyographic (EMG) activity were analyzed. The results showed that quadriceps muscle weakness, produced by repetitive concentric contractions of the knee extensors, induced an improvement of spatiotemporal parameters for patients and healthy subjects. For the patient group, the increase in gait velocity and step length was associated with i) an increase of sagittal hip and knee flexion during the swing phase, ii) an increase of the maximal normalized length of the RF and VL and of the maximal VL lengthening velocity during the pre-swing and swing phases, and iii) a decrease in EMG activity of the RF muscle during the initial pre-swing phase and during the latter 2/3 of the initial swing phase. These results suggest that quadriceps fatigue did not alter the gait of patients with hemiparesis walking with a SKG and that neuromuscular fatigue may play the same functional role as an anti-spastic treatment such as botulinum toxin-A injection. Strength training of knee extensors, although commonly performed in rehabilitation, does not seem to be a priority to improve gait of these patients.

## Introduction

Stiff-knee-gait (SKG) is characterized by a decrease in peak knee flexion during the swing phase of gait and is frequently observed in patients with hemiparesis following stroke [1], [2]. Overactivity of the rectus femoris (RF) muscle related to spasticity causes excessive knee extension moments during swing and has been widely implicated in SKG [3]. This over-activity of the RF is mainly observed in initial pre-swing and may continue in the early and middle swing phases [4], [5], [6], [7]. Spasticity has been defined by Lance (1980) [8] as “a motor disorder characterized by a velocity-dependent increase in the tonic stretch reflex with exaggerated tendon jerks, resulting from hyper-excitability of the stretch-reflex”. Spasticity may be the result of intrinsic modifications of the muscle and/or altered reflex properties [9].

Fatigue is another frequent disabling and persistent symptom in chronic neurological diseases such as stroke [10]. There are several definitions of muscle fatigue in the literature. In this study, fatigue is defined as a reduction in the level of the output force produced by muscles during a sustained activity [11]. To our knowledge, the impact of quadriceps fatigue on SKG has never been assessed in patients with hemiparesis. The literature reports paradoxical results regarding the effects of knee extensor muscle fatigue on the gait pattern in healthy subjects. Most studies found no difference in walking speed and stride length after quadriceps muscle fatigue in young healthy subjects [12], [13] whereas these parameters were improved in the fatigued state in older adults [12]. Results are also contradictory regarding knee biomechanics after a quadriceps fatigue protocol in young healthy subjects, since some studies report an alteration of kinematic and kinetic parameters and others report no changes [13], [14], [15]. These examples clearly show that the real impact of quadriceps fatigue on knee kinematics and kinetics during gait is still under debate. Since gait velocity of stroke patients is strongly dependant on the voluntary strength of the knee extensor muscles [16], [17], we may hypothesized that quadriceps fatigue would alter biomechanical gait parameters.

The relationship between spasticity and muscle fatigue is a major concern in clinical practice but it is still unclear in the scientific literature. Some studies suggest that there is an interaction between neuromuscular fatigue and stretch reflex amplitude (which is exacerbated in spastic patients). Indeed, some results obtained in healthy subjects show that the amplitude of the stretch reflex decreases in the fatigued state [12], [18], [19]. This reduction in stretch reflex response might result from an increase in presynaptic inhibition of Ia afferents and/or a decrease in motoneuronal excitability [12], [18], [19]. Conversely, Biro et al. (2007) [20] showed an enhancement of the gain of the muscle spindle pathways during fatigue when descending drive is low. These results suggest that neuromuscular fatigue may have an effect on spasticity but no study has evaluated this association in patients with neurological disorders such as stroke or multiple sclerosis. It has been possible to estimate level of spasticity during gait for a few years since the development of musculoskeletal models. Several studies have thus begun to explore the relationship between muscle-tendon stretch velocity and electromyography (EMG) activity of spastic muscles [21], [22]. For example, Lampire et al., (2013) [23] showed an increase of maximal RF length and lengthening velocity during the swing phase of gait, one month after injection of botulinum toxin type-A (BoNT-A) in the spastic RF of hemiparetic patients with SKG. They concluded that the improvement of peak knee flexion after BoNT-A injection was mainly due to the decrease in spasticity. These studies suggest that this type of assessment is particularly useful for the estimation of changes in spasticity during gait, after a given treatment.

Therefore, the aim of this study was to evaluate the effects of quadriceps muscle fatigue on biomechanical gait parameters, particularly RF muscle kinematics (length and lengthening velocity) and inappropriate EMG activity, in order to estimate changes in spasticity in stroke patients with SKG caused by RF overactivity. To that end, spatiotemporal parameters, sagittal knee and hip kinematics during swing, RF and VL kinematics and EMG activity were quantified in a population of chronic stroke patients with inappropriate RF activity, before and after an isokinetic fatigue protocol and compared with healthy subjects. We hypothesized that neuromuscular fatigue of the knee extensor muscles would i) alter spatiotemporal and kinematics gait parameters and ii) increase quadriceps muscle activity and RF stretch reflex at the origin of the SKG.

## Materials and Methods

### Subjects

Thirteen patients (8 males and 5 females) with chronic hemiparesis following stroke were enrolled in this study (Table 1, age: 48 (14) years; height: 171 (11) cm, mass: 70 (14) kg). Inclusion criteria of patients: over 18 years old, more than 6 months post stroke (chronic-phase), decrease in peak knee flexion in swing, RF EMG activity in mid swing phase, with a score ≥1+ on Modified Ashworth Scale (MAS) [24] for the quadriceps muscle (tested with the hip in extension), ability to walk independently 10 m without walking aids or assistance, no use of anti-spastic medications 6 months before inclusion and no orthopedic surgery in the last 6 months. In addition, eleven healthy subjects without neurological or musculoskeletal pathologies were recruited (8 males and 3 females; 44 (14) years; 175 (11) cm; 77 (17) kg). This study was approved by the local ethics committee, “Comité de Protection des Personnes Ile de France XI”, and all subjects provided written informed consent prior to participation in any study-specific procedures.

**Table 1:** Demographic characteristics of subjects.

		Hemiparetic patients (N = 13)	Healthy subjects (N = 11)
		Description of populations	
Gender (M/F)		8/5	8/3
Age (years)		48 (14)	44 (14)
Height (cm)		171 (11)	175 (11)
Weight (kg)		70 (14)	77 (17)
Paretic side (R/L)		7/6	–
Time since stroke (months)		84 (47)	–
ABILOCO (logits)		5.00 (1.59)	–
		**Clinical examination**	
**Spasticity**			
MAS	Quadriceps	2[0,2]	–
	Hamstrings	0[0,2]	–
	Triceps surae	1.5[2,2]	–
**Strength**			
MRC scale	Quadriceps	4[4], [5]	–
	Hamstrings	4[3], [4]	–
	Triceps surae	3[1], [3]	–

For the demographic characteristics of hemiparetic patients and healthy subjects, mean (SD) values are presented. For the clinical examination, median values [1^st^; 3^rd^ quartiles] are presented. M = male, F = female, R = right, L = left, MAS = Modified Ashworth Scale, MRC = Medical Research Council.

### Procedure

#### Experimental setup

On their arrival at the laboratory and before the first gait analysis, patients’ locomotion ability was assessed using the ABILOCO questionnaire [25]. Next, patients underwent a clinical neurological evaluation. Spasticity and strength of quadriceps, hamstring and triceps surae muscles were evaluated using the Modified Ashworth Scale (MAS) and Medical Research Council (MRC) scale. Then all subjects were equipped with reflective markers and with surface electrodes. Three gait analyses were carried out by participants i) before the quadriceps fatigue protocol (PRE condition), ii) immediately after (POST_0 condition) and iii) after a 10 min seated rest (POST_10 condition). The duration of the rest period was chosen based on previous studies showing that after a few minutes, patients have already recovered a significant portion of their muscle strength [14], [26].

#### Isokinetic fatigue protocol

A ConTrex-MJ isokinetic dynamometer (Contrex, CMV AG, Dübendorf, Switzerland) which allowed recording of instantaneous isokinetic torque was used for the fatigue protocol. Subjects were seated in the chair of the isokinetic dynamometer in a position of 85° of hip flexion with the lower legs hanging over the edge of the seat. The trunk and the paretic lower leg were stabilized using straps across the chest and the waist, and upper thigh, respectively. The contralateral lower limb was also blocked. The axis of the dynamometer was visually aligned with the knee joint axis, which was defined as a line between the medial and lateral condyles of the femur. The distal attachment of the lower limb to the lever of the dynamometer was 3 cm above the lateral malleolus. The passive range of motion designated as acceptable by the patient was determined and used to set the limits of the isokinetic dynamometer. Before the fatigue protocol, calculation of the limb weight was carried out during passive movements in order to remove the gravitational effects of the limb and attachment from each trial. Next, gravity correction was performed with the ConTrex-MJ software (Contrex, CMV AG, Dübendorf, Switzerland).

Participants performed 8 to 12 sub-maximal concentric knee extension repetitions to familiarize themselves with the task. The paretic lower limb for each patient and the non-dominant lower limb for healthy subjects were fatigued. After the warm-up, each subject performed four maximal voluntary contractions (MVC) of the knee extensors at 60°/s. The highest value of torque was taken as maximal torque (maxMVC). The unilateral voluntary isokinetic fatigue protocol consisted of maximal repetitive concentric contractions of the knee extensor muscles at 60°/sec, with a passive return to the initial knee position at 30°/s [10]. Three series were repeated with one minute rest between each. Each series was stopped when the participants reached three consecutive repetitions below 50% of their maxMVC. No limitations were imposed with regard to the number of repetitions. Strong verbal encouragement was given to the subjects during each contraction. Immediately after the end of the fatigue protocol, subjects were asked to estimate rate of perceived exertion on a 6 to 20 Borg scale [27]. After the fatigue protocol, each participant performed the POST_0 condition gait analysis.

#### Gait analysis

Gait parameters were recorded using 8 optoelectronic cameras (Motion Analysis Corporation, CA, USA) which measured the tridimensional coordinates of 30 reflective markers. Markers were positioned according to the Helen Hays protocol [28]. Nine gait trials were recorded but the three first were removed in order to exclude the adaptation which occurs at the beginning of a gait analysis [29]. Each gait trial was carried out in a 10 m gait corridor. All subjects walked at their self-selected walking velocity. The number of gait cycles recorded was different for the hemiparetic patients and for the healthy subjects (PRE condition: 228 and 95, respectively; POST_0 condition: 212 and 83, respectively; POST_10: condition 199 and 84, respectively). However, in order to make reliable comparison, the number of gait cycles analysed and averaged was significantly similar between the pre and the post fatigue conditions (POST_0 and POST_10), for the two group of subjects. Data were filtered using a fourth-order zero-lag Butterworth low-pass-filter with a cutoff frequency of 6 Hz. Between each gait trial, no rest was allowed and the recording was only stopped between trials while the subjects turned around. The position of the reflective markers was maintained during the whole experiment to limit bias relative to the repositioning of the markers. Ground reaction forces were measured synchronously with the kinematic data using two force-plates (AMTI, Watertown, MA, USA, sampling frequency 1000 Hz) staggered along the walkway.

#### Electromyographic assessment

Electromyographic (EMG) activities of the RF and vastus lateralis (VL) of the paretic limbs for patients and of the non-dominant leg for healthy subjects were recorded during the gait trials and during each isokinetic session. Two bipolar surface electrodes with built-in pre-amplification (MA311, Motion Lab Systems, LA, USA) were placed directly on the skin, according to SENIAM recommendations [30]. The occurrence of crosstalk (particularly RF and vastus intermedius) cannot be completely excluded, especially during gait that require synergic activation of more than one muscle [31]. The EMG sensors were composed of two circular dry button electrodes (stainless steel) with double-differential preamplifiers. The two active electrodes measured 12 mm in diameter and the inter-electrode distance was 17 mm. All EMG signals were sampled at 1000 Hz, pre-amplified with a gain of 20. Before processing the EMG signal, all signals were band-pass filtered between 3.5 and 350 Hz [32].

### Data Analysis

#### Fatigue assessment

Only data (torque and EMG) from the constant velocity portion of each concentric trial were analyzed because, during acceleration and deceleration phases, data are strongly affected by inertial forces [33]. All isokinetic and EMG data were exported to text files and processed using custom algorithms developed in MatLab version 9.0 (The MathWorks, Inc., MA, USA). The repetitions carried out before the subject to reached 50% of maxMVC were averaged for the 3 sets of fatigue. Peak torque for each repetition and each subject were normalized by his maxMVC. Next, the index of fatigue was calculated as the rate of decline in normalized peak torque represented by the slope of the linear regression, beginning with the highest value of the first 5 consecutive repetitions and ending with the last 5 repetitions [34]. This fatigue index calculation has previously shown to be valid in patients with neurological disorders [35], [36]. The RMS values of RF and VL muscles were calculated over a 500 ms period around the occurrence of peak torque (i.e. 250 ms before and 250 ms after) for each repetition and each subject. In addition, the average normalized maximum peak torque and the root mean square (RMS) values of RF and VL EMG was calculated for the first three and the last three repetitions.

#### SKG assessment

Spatiotemporal and sagittal joint kinematic parameters were computed for each gait cycle, using OrthoTrack 6.5 software (Motion Analysis Corporation, CA, USA). The following spatiotemporal parameters were calculated: gait velocity, step length and cadence. The sagittal hip and knee angles during the swing phase and angular velocity at toe-off were computed for the fatigued limb of both groups [37]. Only the sagittal kinematic plane was evaluated to identify the deviations most frequently described in hemiparetic gait [2], [38].

#### Estimation of RF spasticity during gait

Normal RF muscle activity occurs during the latter half of pre-swing (from final double support to toe-off) and the first third of initial swing. RF muscle activity is considered inappropriate if it occurs during the first half of pre-swing, the latter 2/3 of initial swing, or is present during any portion of mid-swing [2], [39]. Hence, inappropriate EMG activity was evaluated during these 3 periods. Mean values of EMG activity of RF and VL muscles were calculated for each sub-phase of gait and each subject. The linear envelope of the EMG signals was calculated according to Shiavi’s method (1987) [40]. The amplitude of the linear envelope was normalized with respect to the maximum value recorded across all gait trials in the PRE condition and Mean Amplitude Value (MAV) was calculated as the mean value of the normalized linear envelope over the time interval.

SIMM, (MusculoGraphics, Inc., Santa Rosa, California, USA) modeling software (Delp et al., 1990) was used to calculate the length and lengthening velocity of the musculo–tendinous complex of RF and VL muscles. For a detailed description of the method, see Lampire et al. (2013) [23]. Normalized maximal RF and VL lengths (maxNL) and maximal RF and VL lengthening velocity (maxLV) were calculated during the pre-swing and swing phases.

### Statistical Analyses

Kolmogorov-Smirnov tests were conducted before the statistical analysis and confirmed that data were normally distributed. Due to the large difference in baseline values between the two groups, a one-factor ANOVA with repeated measures was used to analyze differences between the three conditions (PRE vs. POST_0 vs. POST_10) for each group separately (hemiparetic patients and healthy subjects) for each gait parameter. Post hoc analysis was performed using the Bonferroni post hoc multiple comparison test. For each parameter where we observed a significant change over time, the percentage of changes for this parameter was compared between the groups using a Student’s t test. Comparisons of the several fatigue protocol parameters between the two groups were performed with a Student T-test. The significance level was maintained at p<0.05, with Bonferroni adjustments used as appropriate. Values were expressed as mean ± standard deviation. Statistical analysis was performed using Statistica 7 (StatSoft, Inc., Tulsa, OK, USA).

## Results

### Fatigue Protocol (Table 2)

Post-fatigue, rate of perceived exertion (RPE) on a 6 to 20 point Borg scale was not significantly different between the two groups (Table 2). The maxMVC was significantly higher for healthy subjects compared to patients with hemiparesis (respectively, 173.9 (62.2) N.m and 78.3 (33.9) N.m, p<0.001). The number of repetitions to reach 50% of maxMVC, was not different between the two groups (64.7 (22.7) for hemiparetic and 54.8 (17.6) for healthy subjects, p = 0.556). The magnitude of the slope value (fatigue index) was −0.37 (0.30) and −0.62 (0.69) N.m/rep respectively for the hemiparetic and healthy groups (Table 3), but the difference between the two groups was not significant. The RMS amplitude of the RF muscle was significantly lower in the last three contractions than in the first three, for both the hemiparetic and healthy groups (respectively, 240.6 (186.6) to 211.2 (168.3) μV, p = 0.027 and 576.2 (220.6) to 438.5 (142.2) μV, p = 0.028). The RMS amplitude of the VL muscle was not modified by the fatigue protocol (Table 2).

**Table 2:** Fatigue protocol parameters.

	Hemiparetic patients (N = 13)		Healthy subjects (N = 11)	
	Fatigue protocol			
maxMVC (N.m)	78.3 (33.9)		173.9 (62.2)†	
Mean total repetitions	64.7 (22.7)		54.8 (17.6)	
Fatigue Index (N.m/rep)	−0.37 (0.30)		−0.62 (0.69)	
RPE after the fatigue protocol	12 [11]; [13]		12 [12]; [15]	
	**First 3 repetitions**	**Last 3 repetitions**	**First 3 repetitions**	**Last 3 repetitions**
RMS RF (μV)	240.6 (186.6)	211.2 (168.3)*	576.2 (220.6)	438.5 (142.2)*
RMS VL (μV)	378.7 (231.8)	354.1 (192.5)	588.1 (219.8)	509.9 (243.5)

For the fatigue protocol parameters mean (SD) values are presented. RPE (rate of perceived exertion) values are expressed as median values [1^st^; 3^rd^ quartiles]. EMG = electromyography, RF = rectus femoris, VL = vastus lateralis.

† Significant difference between groups (p<0.05).

*Significant difference between the beginning and the end of the fatigue protocol (p<0.05).

**Table 3:** Spatio-temporal, kinematic and EMG parameters during gait.

		Hemiparetic patients (N = 13)			Healthy subjects (N = 11)		
		PRE	POST	POST_10	PRE	POST	POST_10
**Spatio-temporal parameters**							
Velocity (m/s)		0.68 (0.19)	0.75 (0.22)†	0.78 (0.21)*	1.32 (0.22)	1.41 (0.20)†	1.42 (0.23)*
Step length (m)		0.46 (0.08)	0.49 (0.09)†	0.50 (0.08)*	0.67 (0.06)	0.68 (0.08)	0.68 (0.08)
Cadence (step/min)		89.4 (15.5)	92.5 (15.8)†	94.9 (16.6)*	116.0 (10.1)	122.2 (11.4)†	120.8 (11.0)*
**Joint kinematics**							
Peak hip flexion in swing (°)		36.3 (9.2)	38.8 (8.9)†	37.7 (9.4)*	33.6 (6.8)	33.8 (4.9)	34.4 (6.5)
Peak knee flexion in swing (°)		39.0 (13.8)	43.8 (17.1)†	43.0 (16.7)*	60.2 (4.9)	61.3 (4.6)	61.1 (4.2)
Knee angular velocity at toe off (°/s)		180.9 (96.2)	193.6 (94.2)†	190.5 (98.7)*	314.7 (37.2)	300.5 (39.4)	314.2 (41.4)
Hip angular velocity at toe off (°/s)		101.9 (49.0)	116.4 (54.1)†	114.4 (55.4)*	121.0 (22.1)	130.8 (23.3)	129.7 (22.0)
**Muscle kinematics**							
Pre-swing	MaxNL RF (mm)	12.9 (8.2)	14.6 (8.4)†	16.4 (9.1)*	21.8 (12.7)	22.4 (13.8)	22.4 (13.4)
	MaxLV RF (m/s)	0.15 (0.09)	0.16 (0.09)†	0.16 (0.11)*	0.24 (0.04)	0.25 (0.02)	0.25 (0.02)
	MaxNL VL (mm)	16.9 (7.1)	17.1 (6.6)	18.4 (7.0)	18.6 (8.2)	19.4 (8.3)	18.6 (7.9)
	MaxLV VL (m/s)	0.20 (0.11)	0.21 (0.11)†	0.22 (0.12)*	0.25 (0.09)	0.26 (0.09)	0.26 (0.09)
Swing	MaxNL RF (mm)	17.0 (13.4)	20.0 (12.6)†	20.1 (13.9)*	37.6 (8.9)	38.1 (8.6)	38.4 (8.6)
	MaxLV RF (m/s)	0.12 (0.10)	0.14 (0.09)†	0.13 (0.10)*	0.24 (0.03)	0.24 (0.03)	0.25 (0.03)
	MaxNL VL (mm)	28.3 (10.4)	30.7 (10.8)†	30.2 (10.8)*	40.3 (16.3)	40.9 (16.4)	40.6 (16.3)
	MaxLV VL (m/s)	0.18 (0.22)	0.22 (0.13)†	0.21 (0.13)*	0.27 (0.10)	0.28 (0.09)	0.28 (0.09)
**EMG activity**							
Initial Pre-swing	EMG RF (%)	23.9 (8.9)	15.2 (8.7)†	16.6 (8.3)*	15.8 (5.0)	17.6 (8.4)	16.4 (10.9)
	EMG VL (%)	10.8 (6.6)	8.4 (7.5)	9.4 (7.4)	8.9 (4.2)	8.7 (4.5)	6.3 (3.2)
Normal activity	EMG RF (%)	29.1 (10.3)	21.9 (11.1)	25.3 (14.6)	26.0 (11.8)	27.7 16.1)	26.1 (15.6)
	EMG VL (%)	8.9 (6.5)	7.9 (6.0)	9.1 (6.2)	10.9 (6.3)	15.6 (17.0)	11.3 (11.8)
Latter 2/3 of initial swing	EMG RF (%)	31.7 (14.8)	23.9 (14.3)	25.5 (16.8)	22.9 (11.4)	29.7 (19.7)	19.9 (12.1)
	EMG VL (%)	8.5 (6.7)	6.4 (4.1)	7.1 (4.1)	10.5 (5.0)	10.6 (7.4)	11.0 (14.2)
Mid-swing	EMG RF (%)	24.3 (10.7)	18.6 (10.8)	22.7 (17.6)	14.6 (4.0)	13.1 (3.9)	11.3 (3.6)
	EMG VL (%)	11.5 (7.7)	8.5 (5.8)	9.1 (4.4)	7.9 (3.7)	6.9 (4.0)	5.8 (3.3)
Terminal swing	EMG RF (%)	26.2 (9.6)	20.5 (11.1)	21.5 (10.3)	27.3 (5.3)	23.1 (7.8)	21.52 (8.1)
	EMG VL (%)	22.4 (9.5)	15.5 (10.2)	22.6 (15.1)	26.0 (13.0)	23.7 (16.4)	20.6 (14.6)

Mean (SD) spatio-temporal parameters, joint kinematics, muscle kinematics and EMG for the fatigued limb in PRE condition, POST_0 condition and POST_10 condition. MaxNL = Normalized maximal length; MaxLV = maximal lengthening velocity; EMG = electromyography, RF = rectus femoris, VL = vastus lateralis.

† Significant difference between PRE and POST_0 (p<0.05).

*Significant difference between PRE and POST_10 (p<0.05).

### SKG Assessment (Table 3)

For the patients with hemiparesis, gait velocity, step length and cadence of the fatigued limb were significantly greater in POST_0 and POST_10 compared with PRE (p<0.001).

**Figure 1:**
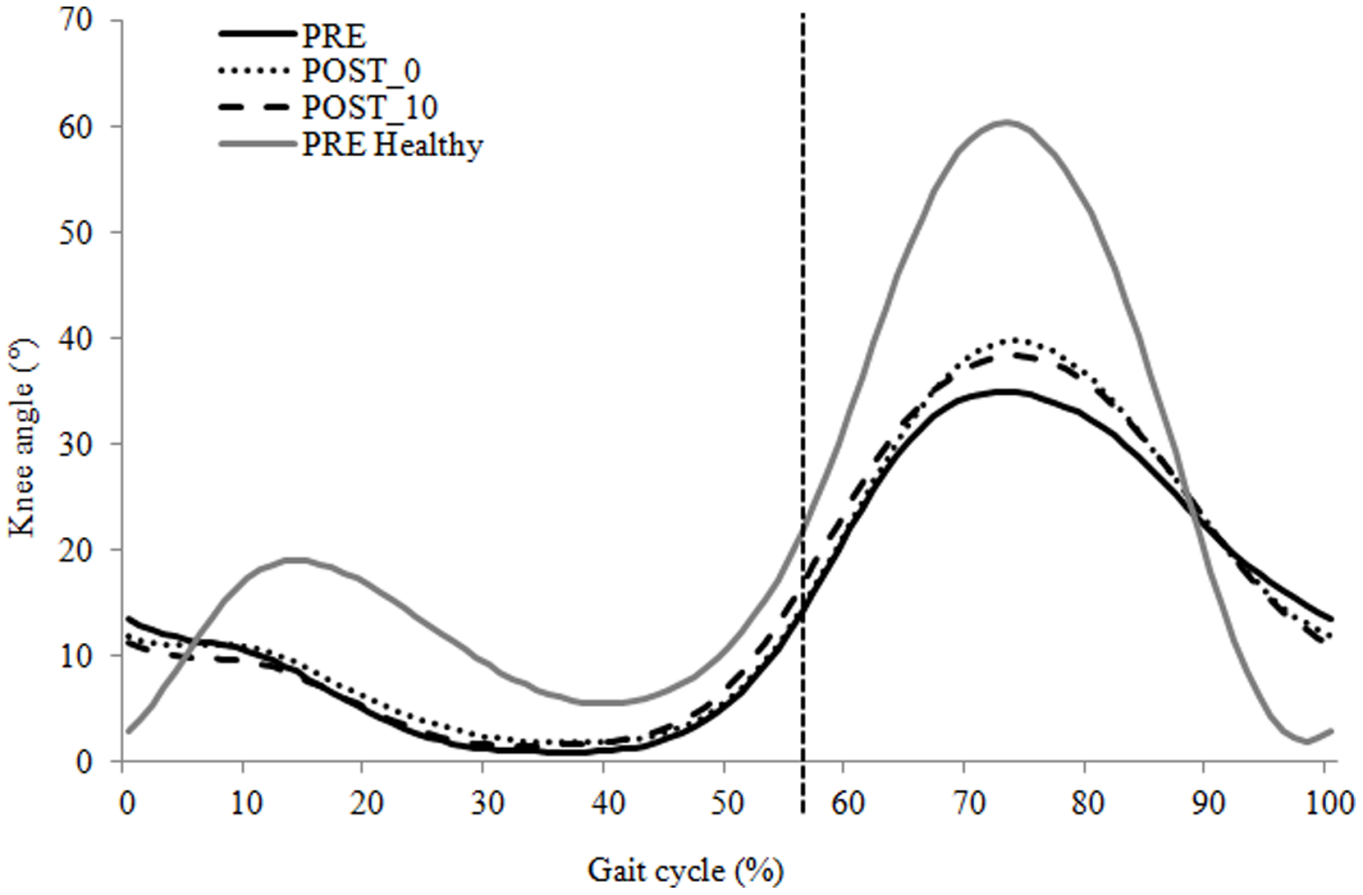
Mean sagittal-plan knee joint angle for the hemiparetic group and the healthy group. The solid black line indicates the PRE condition for the hemiparetic group walking at a mean spontaneous gait velocity of 0.68/s, the dotted black line indicates the POST_0 condition for the hemiparetic group walking at a mean spontaneous gait velocity of 0.75 m/s and the dashed black line indicates the POST_10 condition for the hemiparetic group waking at a mean spontaneous gait velocity of 0.78 m/s. The solid gray line indicates the PRE condition for the healthy group walking at a mean spontaneous gait velocity of 1.32 m/s. The vertical solid line represents the beginning of the swing phase of gait.

Peak hip and knee flexion angles during swing were significantly greater in POST_0 and POST_10 compared with PRE (p = 0.04 and p = 0.03, respectively) (Figure 1). Hip and knee angular velocities at toe-off were significantly greater in POST_0 and POST_10 compared with PRE (p = 0.002 and p = 0.04, respectively). There were no significant changes in parameters between POST_0 and POST_10.

For healthy subjects, gait velocity and cadence were significantly greater in POST_0 and POST_10 conditions compared with PRE (p<0.001). No differences were observed between group for the percentage of changes of these two gait parameters appearing between PRE and POST_0 (p = 0.16) and between PRE and POST_10 (p = 0.06). None of the other parameters were significantly modified by the fatigue protocol.

### Estimation RF Spasticity during Gait (Table 3)

**Figure 2:**
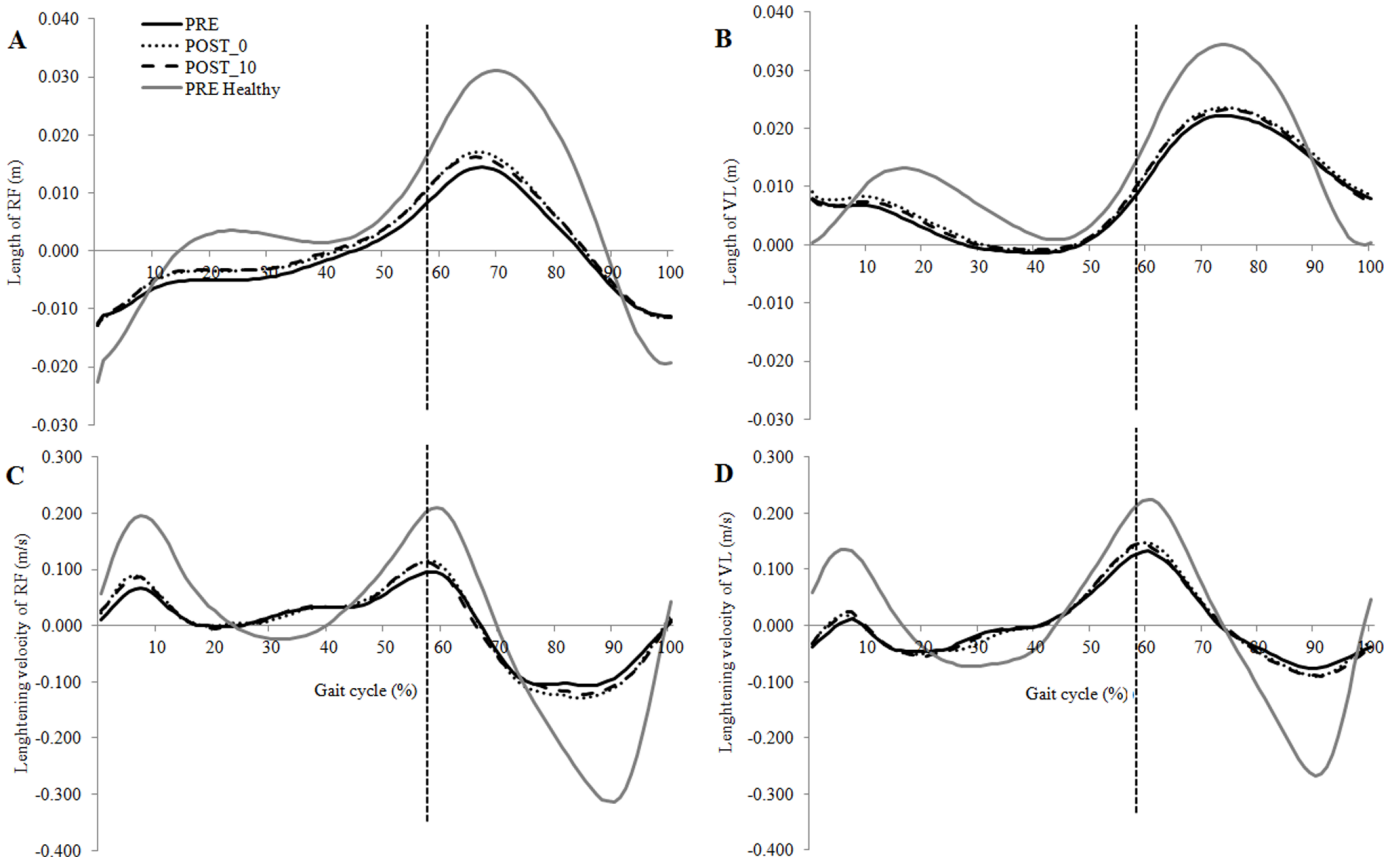
Mean normalized rectus femoris (A) and vastus lateralis length (B), and mean lengthening velocity curve of rectus femoris (C) and vastus lateralis (D). The solid black line indicates the PRE condition for the hemiparetic group walking at a mean spontaneous gait velocity of 0.68/s, the dotted black line indicates the POST_0 condition for the hemiparetic group walking at a mean spontaneous gait velocity of 0.75 m/s and the dashed black line indicates the POST_10 condition for the hemiparetic group walking at a mean spontaneous gait velocity of 0.78 m/s. The solid gray line indicates the PRE condition for the healthy group. The vertical solid line represents the beginning of the swing phase of gait. RF = rectus femoris; VL = vastus lateralis.

For the patients with hemiparesis, maxNL of RF (p = 0.003) and maxLV of VL (p = 0.005) in pre-swing were greater in POST_0 and POST_10 compared with PRE. In swing, maxNL of RF (p = 0.004), maxNL of VL (p = 0.004) and maxLV of VL (p = 0.003) were greater in POST_0 and POST_10 compared with PRE (Figure 2). The MAV of RF was lower during initial pre-swing (p = 0.003) in POST_0 and POST_10 compared with PRE. No parameters were significantly modified between POST_0 and POST_10.

For the healthy subjects, no change occurred in any muscle kinematic parameter or in the EMG activity.

## Discussion

The aim of this study was to evaluate the effects of quadriceps muscle fatigue on biomechanical gait parameters, in hemiparetic patients with SKG. The key finding of this study was that fatigue, produced by repetitive concentric contractions of the knee extensors, induced an improvement in the spatiotemporal parameters of both patients and healthy subjects. For the patient group, the increase in gait velocity and step length was associated with i) an increase of sagittal hip and knee flexion during the swing phase, ii) an increase of the maximal normalized length of the RF and VL and of the maximal VL lengthening velocity during the pre-swing and swing phases, and iii) a decrease in EMG activity of the RF muscle during the initial pre-swing and the latter 2/3 of initial swing phases. These results did not confirm our initial hypothesis.

The number of repetitions to reach 50% of the baseline maxMVC and fatigue index was not statistically different between the two groups, despite different mean values. These results are in contradiction with the literature since, in a recent review, Knorr et al. (2012) [10] showed that muscles of the paretic limb are more fatigue-resistant than muscles of the non-paretic limb or than muscles of healthy subjects. Muscle weakness, deficit of voluntary muscle activation, disordered motor control and/or greater reliance on type I fibers have been proposed to explain the lower peripheral fatigue rate in the paretic limb [10], [41]. It seems important to note that the EMG activity (RMS amplitude) of RF muscle decreases, for both groups, between the beginning and the end of the fatiguing exercise when it is not modified for the VL muscle. This result confirm previous studies showing that after an isokinetic fatigue protocol (knee-extensions), fatigue is more pronounced in the RF muscle than in the vastii [42]. Indeed, the bi-articular RF and mono-articular vastii play different functions during seated isokinetic knee-extensions tasks [43].

The present study suggests that specific fatigue of quadriceps muscle improves spatiotemporal gait parameters of both patients and healthy subjects. It should be noted that because of the inclusion criteria (ability to walk independently 10 m without walking aids or assistance), the group of patients presented a good locomotion capacity, explaining the high score on ABILOCO scale. Hence, the results reported in this study are could be only specific to a population of patients with the same clinical characteristics. Our results are consistent with previous studies which highlighted an improvement in gait velocity and step length, in the fatigued state, in older adults [12] and in patients with hemiparesis [44]. However, in the present study, the increase in gait velocity after fatigue occurred via different mechanisms in the healthy subjects and patients, since patients increased both cadence and step length, whereas healthy subjects only increased their cadence. The increase in cadence in the fatigued state has previously been observed in healthy subjects [45]. It is recognized that balance is affected by lower limb fatigue [46]. The concomitant increase in gait speed and cadence may contribute to a better medio-lateral stability due to the decrease in medio-lateral displacement of the center of mass [47], [48]. This seems to be a strategy to compensate for impaired balance in the fatigued state.

We can hypothesize that the improvement in step length in the patient group is directly linked with changes in knee and hip flexion of the fatigued limb during the swing phase and thus with the increase in knee and hip angular velocities at toe-off. In order to better understand improvement of walking capacity after fatigue, changes in the non-fatigued limb were also evaluated. Because we observed no significant changes, these results are not presented in this manuscript. After fatigue, the increase in peak hip flexion in the swing phase may be surprising. Indeed, the RF is a bi-articular muscle which acts as hip flexor and knee extensor. Therefore, RF muscle weakening might decrease hip flexion. Granacher et al. (2010) [12] found that the increase in gait velocity after a fatigue protocol induced by isokinetic contractions could be associated with an increase in hip flexion in older adults. They proposed that older adults may have compensated for the fatigue induced decrease in knee extensor strength by increasing hip flexor power during walking. This strategy, frequently observed in older adults to compensate for muscle deficits [49], [50], may explain the surprising increase in peak hip flexion, in the swing phase for the patient group. In contrast, this did not occur in healthy subjects since their muscle function is normal.

The analysis of EMG signals and muscle kinematics explains the increase in knee flexion during the swing phase. The EMG activity of the RF muscle during the initial pre-swing phase was significantly lower in the fatigued state, while maxNL and maxLV of RF and VL during the swing phase were significantly higher. During the double support phase of gait, the leg prepares for toe-off, the hip reaches maximum extension and the knee begins to flex. Excessive and prolonged activity of the RF muscle in the pre-swing phase could be induced by the excessive response to muscle stretch, leading to the inappropriate knee extension moment observed in the SKG pattern [51]. In the present study, the fatigue protocol mainly modified the excessive response to RF stretch during the initial pre-swing phase. From these results, it may be hypothesized that the repetition of maximal isokinetic contractions until fatigue is reached affects the stretch reflex loop of this muscle. Granacher et al., (2010) [12] also observed a decline of the functional reflex amplitude in tibialis anterior during gait perturbations, after a similar isokinetic fatigue protocol. A possible explanation could relate to group III and group IV free nerve endings which are sensitive to muscle metabolite accumulation [52], [53]. It was reported that the inhibitory effect of group III and group IV muscle afferents reduces the discharge of gamma motoneurons and influences Renshaw inhibition [54]. Their projections on inhibitory interneurones could reduce the excitatory input by presynaptic inhibition of the group-Ia afferents [55], [56], predominantly in the extensor muscles [57]. Moreover, Marque et al., (2001) [58] reported that spasticity in the quadriceps muscle can be related to facilitation of the spinal reflex via group-II muscle spindles. Since group-II muscle afferents project on inhibitory interneurones [59], we could hypothesize that metabolite accumulation affecting group III-IV muscle afferents may cause presynaptic inhibition of the group-Ia afferents, and reduce the recruitment of α-motoneurons during the quadriceps stretch. Therefore, the group III and group IV muscle afferents could mediate a reflex which may modulate motoneuron firing rate in a fatigued state [60].

Finally, it seems important to note that the kinematic and EMG improvements, observed in the present study, are very similar to those reported in several studies which evaluated the effects of BoNT-A in the RF to decrease SKG in patients with hemiparesis [6], [21], . For example, Lampire et al. (2013) [23] showed that after a BoNT-A injection in the RF of patients with hemiparetic SKG, the increase in knee flexion during swing was correlated with the increase of maxNL of the RF, and associated with an increase in its maximal lengthening velocity. This seems to suggest that the quadriceps fatigue protocol induced a decrease in spasticity which was at the origin of the observed gait improvement, mainly walking velocity. Several studies have shown that the increase of the gait velocity is associated with an increase in knee flexion velocity at toe-off and an increase in peak knee flexion during swing phase of the gait cycle in stroke patients [64], [65]. Therefore, we may hypothesize that the kinematic changes observed in the present study are the result of the velocity increase. To answer this question, correlations between the percentage of improvement of RF length or RF maxLV induced by fatigue and the percentage of improvement of the walking velocity have been calculated. No significant correlation was observed. This suggests that the kinematic changes observed in this study are not due to an increase in walking velocity.

The decrease of muscle overactivity after BoNT-A is due to its action on the neuromuscular junction [58] and on the motoneuron γ-intrafusal spindle synapse [66], [67], [68], [69]. Although the BoNT-A injection and the present fatigue protocol seem to have a similar effect on SKG, our study cannot determine whether the physiological causes of these mechanical changes are similar. Therefore, further neurophysiological explorations must be conducted in order to precisely determine the origins of the fatigue induced by this protocol and to estimate the long-term effects of this kind of exercise on spasticity during gait. However, our results may have important implications for designing strength training programs. In clinical practice, spasticity of lower limbs, in patients with hemiparesis, could be managed by combining BoNT-A injection and resistance training. However, the results of the present study clearly show that quadriceps weakness did not alter the gait of hemiparetic patients with SKG. Although the present results were observed after a single session of muscle exertion, they pose the question of the interest of the knee extensors strengthening to improve gait in these patients [70].

This study has several limitations. First, the small number of patients (n = 13) and of healthy subjects (n = 11) included could potentially limit the statistical power of the results. Secondly, our group of patients is relatively young and presents high locomotion ability. So the sample may not be representative of the stroke population. These limitations mean that results must be interpreted with caution and further studies will be conducted in order to confirm our results.

## Conclusion

In conclusion, our results clearly show that a specific quadriceps fatigue improved gait velocity in patients with hemiparetic SKG and in healthy subjects. For the patient group, the increase of gait velocity and step length was associated with improvements of peak hip and knee flexion during the swing phase. The decrease in inappropriate activity of RF muscle, initially due to the exaggeration of the stretch reflex in the initial pre-swing phase, seems to show that the increase in knee flexion during swing was due to a decrease in spasticity. With regard to these results, it seems that neuromuscular fatigue may play the same functional role as anti-spastic treatments such as BoNT-A injections during gait of patients with hemiparesis.
